# Enhancing UHPC Tensile Performance Using Polystyrene Beads: Significant Improvements and Mechanisms

**DOI:** 10.3390/ma17112479

**Published:** 2024-05-21

**Authors:** Lang-Kuo Guo, Li-Biao Chen, Zhi-Wei Chen, Jun-Yan Wang

**Affiliations:** 1 School of Materials and Engineering, Tongji University, Shanghai 201804, China; 2 Key Laboratory of Advanced Civil Engineering Materials, Tongji University, Ministry of Education, Shanghai 201804, China; 3 Fujian Expressway Group Co., Ltd., Nanping 354300, China

**Keywords:** UHPC, tensile strength, artificial flaw, X-ray CT, fiber orientation, polystyrene beads

## Abstract

This study investigates utilizing spherical polystyrene (PS) beads as artificial flaws to improve ultrahigh-performance concrete (UHPC) tensile performance using a uniaxial tensile test and explains the corresponding mechanisms by analyzing the internal material structure of UHPC specimens with X-ray CT scanning. With a hooked steel fiber volume fraction of 2%, three PS bead dosages were employed to study tensile behavior changes in dog-bone UHPC specimens. A 33.4% increase in ultimate tensile strength and 174.8% increase in ultimate tensile strain were recorded after adding PS beads with a volume fraction of 2%. To explain this improvement, X-ray CT scanning was utilized to investigate the post-test internal material structures of the dog-bone specimens. AVIZO software was used to analyze the CT information. The CT results revealed that PS beads could not only serve as the artificial flaws to increase the cracking behavior of the matrix of UHPC but also significantly optimize the fiber orientation. The PS beads could serve as stirrers during the mixing process to distribute fiber more uniformly. The test results indicate a relationship between fiber orientation and UHPC tensile strength.

## 1. Introduction

Ultrahigh-performance concrete (UHPC) is a fiber-reinforced cementitious material widely used in civil engineering structures due to its ultrahigh strength and durability [1]. The incorporation of steel fibers in UHPC results in significantly enhanced tensile performance compared to normal concrete, showing even pseudo-strain-hardening behavior under tension [2,3,4].

Existing methods to improve the tensile properties of UHPC include increasing the fiber content, increasing the fiber aspect ratio, using anisotropic fibers, and modifying the fiber surface [2,5,6,7,8,9,10]. Most of these methods improve the tensile mechanical properties of UHPC by adjusting the fiber parameters, which has some shortcomings. Steel fibers usually account for more than 40% of UHPC’s total cost [11,12]. In order to further reduce the cost of concrete structures, scholars have made efforts in many aspects [13], and further reducing the cost of UHPC is conducive to promoting the application of UHPC in engineering. Increasing the steel fiber dosage can significantly increase costs, and an excessively high steel fiber content and aspect ratio can cause fiber agglomeration, which can adversely impact the mechanical properties of UHPC [14].

An engineered cementitious composite (ECC) is a fiber-reinforced cementitious material with an ultimate tensile strain of several percent. ECCs can exhibit pseudo-strain-hardening behavior under tension, accompanied by multiple-cracking behavior [15]. UHPC and ECCs are both categorized as fiber-reinforced cementitious materials. The methods used to enhance ECC tensile performance are worth considering as potential approaches to improve UHPC tensile performance. Introducing artificial flaws into the matrix to improve the uniaxial tensile mechanical properties is widely accepted in ECCs [15,16,17,18,19,20,21,22]. Victor Li et al. studied the effects of introducing artificial flaws into an ECC matrix on the tensile performance of ECCs in 2004 [21]. Their results showed that adding a lightweight aggregate (with a volume fraction of 0.07%) increased the ultimate tensile strength of the ECC from 6.40 MPa to 6.82 MPa and the ultimate tensile strain from 0.38% to 2.48%.

**Figure 1:**
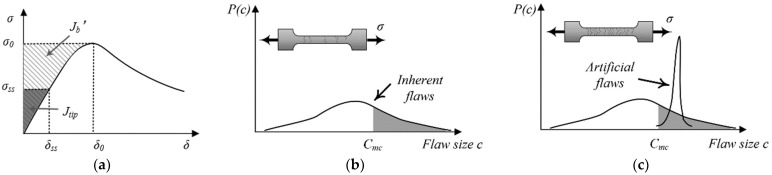
(**a**) A typical σ(δ) curve for a strain-hardening material; (**b**) the size distribution of inherent flaws in a UHPC matrix; (**c**) the size distribution of the superimposed artificial flaws.

Pseudo-strain-hardening behavior accompanied by multiple-cracking behavior implies high levels of ductility, energy absorption capacity, and toughness [23]. To achieve strain hardening, steady-state cracking must occur, and two basic conditions must be satisfied to realize strain-hardening and multiple-cracking behaviors. The first condition is steady-state cracking, wherein the fracture energy of the matrix ($J_{tip}$) needs to be lower than the complementary energy of the bridging stress ($J_{b}\prime$), as shown in Figure 1a. Prior to the propagation of a flat crack, the initiation of a microcrack is influenced by the applied load. However, the applied load must be within the fiber-bridging capacity, meaning that the first-crack strength ($\sigma_{c}$) of the matrix should not exceed the maximum fiber-bridging stress ($\sigma_{0}$). These two conditions are expressed in Equations (2) and (3) [21], as depicted in Figure 1a.

(1) $$J_{tip}=\frac{K_{m}^{2}}{E_{m}}$$

(2) $$J_{tip}\leq \sigma_{0}\delta_{0}-\int_{0}^{\sigma_{0}}\sigma \left(\delta \right)d\delta \equiv J_{b}\prime$$

(3) $$\sigma_{c}\leq \sigma_{0}$$

Assuming that steady-state cracking is initiated by a critical flaw on the section normal to the maximum principal stress, there must be sufficiently large flaws present so that the matrix cracking strength ($\sigma_{c}$) is lower than the peak bridging stress ($\sigma_{0}$) (Equation (3)). Marshall et al. [24,25] demonstrated that $\sigma_{c}$ does not decrease indefinitely with flaw size but reaches a lower bound at the steady-state cracking stress ($\sigma_{ss}$). Equations (2) and (3) ensure the occurrence of multiple cracking, whereby the number of cracks depends on the size and spatial distribution of flaws. To restrict crack activation before reaching the critical stress $\sigma_{0}$ and contribute to multiple cracking, a lower bound critical flaw size $C_{mc}$ is set. Only flaws larger than $C_{mc}$ can be activated and participate in multiple cracking. Therefore, a sufficient number of large flaws must be present in the matrix to achieve saturated multiple cracking. $C_{mc}$ represents the size of an equivalent Griffith crack that has the same critical stress $\sigma_{0}$ for propagation as a real crack. Matrix toughness also determines $C_{mc}$ [25]. Figure 1b,c illustrate the concept of tailoring the matrix flaw size distribution to achieve saturated multiple cracking [26]. Artificial flaws larger than $C_{mc}$ are introduced and combined with the natural flaw system. Since the cracking strength is limited by the steady-state stress, larger flaws do not significantly influence the cracking strength. Therefore, a narrow size distribution is preferred for artificial flaws, as shown in Figure 1c.

Introducing flaws into the UHPC matrix to improve its uniaxial tensile performance is rarely mentioned in previous studies. Su-Tae Kang et al. [27] studied the combined effects of artificial flaws and fiber hybridization on the mechanical properties of UHPC. Plastic particles with an average size of 3.6 mm were added into the UHPC matrix as artificial flaws. Their results showed that compared with a control group, plastic particles have a minor impact on the ultimate tensile strength and tensile strain capacity. To enhance the tensile properties of UHPC while preserving its compressive strength, higher demands have been imposed on the size, physical properties, and introduction method of flaws. Existing research has rarely mentioned a significant improvement in the tensile performance of UHPC through the introduction of artificial flaws into the matrix. These kinds of approaches and corresponding mechanisms remain to be further investigated.

X-ray CT scanning, as a nondestructive, efficient, and high-resolution technology, is widely used for analyzing the internal microstructure of cement concrete [28,29,30]. By utilizing CT scanning, researchers [16,31,32,33] have obtained reliable analysis results regarding flaw structures and fiber distributions in UHPC. Cong Lu et al. [16] employed X-ray CT to investigate the correlation between cracking strength and flaw distribution in ECCs and identified the dimensions of pre-existing flaws as the main influencing parameters. Jussi-Petteri et al. [32] analyzed the orientation of short fibers in steel fiber-reinforced concrete (SFRC) through X-ray CT. The fiber orientation information was obtained and qualitatively assessed. Previous studies [34,35] have shown that compared with conventional imaging, which can only analyze fiber distribution and fiber number in a limited cross-section area, the fiber distribution information obtained with a CT scan can be evaluated on the scale of the entire CT scan sample, which has better accuracy [36,37,38]. CT scanning technology can efficiently obtain information on internal material structures, thus elucidating the changes in mechanical properties.

This paper investigates the feasibility of introducing artificial flaws into a UHPC matrix to improve the uniaxial tensile properties of UHPC and explain the corresponding mechanisms. Based on pre-experiments and the available literature, spherical polystyrene (PS) beads with an average diameter of 4 mm were introduced into the UHPC matrix as artificial flaws, and three PS bead volume fractions were selected. The uniaxial tensile tests were conducted to evaluate the tensile performance of UHPC specimens with different PS bead volume fractions. To explain the changes in uniaxial tensile mechanical properties, X-ray CT scanning was performed on the uniaxial tension specimens. PS beads and steel fibers were reconstructed in three dimensions, and their spatial distributions were assessed. A mechanism for PS beads improving UHPC uniaxial tensile performance was proposed, and the interaction between PS beads and steel fibers was explained.

## 2. Materials and Methods

### 2.1. UHPC Mixture and Specimen Preparation

**Figure 2:**
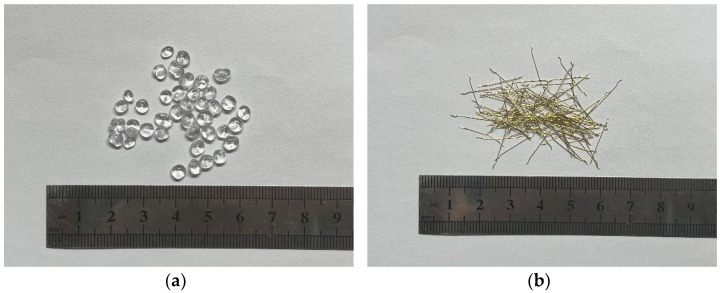
Polystyrene (PS) beads and steel fiber used in the mixer: (**a**) PS beads; (**b**) hooked steel fiber.

The UHPC matrix components for all specimens in this study are listed in Table 1. The study utilized 52.5R cement as the primary cementitious material. As illustrated in Figure 2a, the certain hydrophobic polystyrene (PS) beads, having an average diameter of 4 mm and a density of 1010 kg/m^3^, was selected to be incorporated into the UHPC matrix after preliminary experiments. The polystyrene is composed of styrene monomers (C_6_H_5_CH=CH_2_), and the main functional group in styrene is the aromatic benzene ring (C_6_H_5_-). Due to the weak bond between the PS beads and UHPC matrix, the PS beads can be considered as artificial flaws when subjected to tensile loading. Hooked steel fibers with brass coating, as depicted in Figure 2b, were employed, and detailed information of the steel fibers is listed in Table 2.

**Figure 3:**
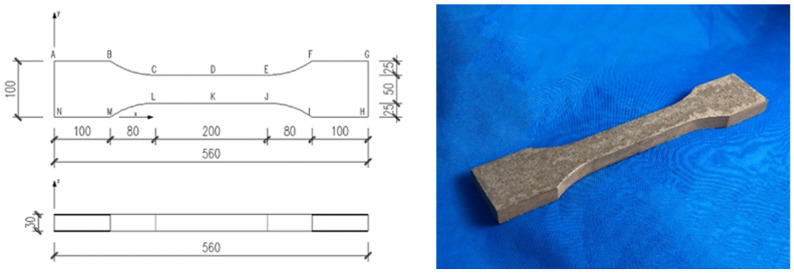
Dog-bone specimen for uniaxial tensile test (in millimeters).

The control group in this study, denoted as F2.0, does not include PS beads. The term “PS” refers to the mixture incorporating PS beads. The volume fraction of steel fibers for both the control group and PS groups was set at 2.0%. The PS groups were further divided into subgroups containing PS beads with volume fractions of 1%, 2%, and 4%. The dimensions of the uniaxial tensile specimens are presented in Figure 3. For each group, the specimens were prepared as follows: (1) three dog-bone uniaxial tensile specimens following the recommendation issued by MCS-EPFL [39] and (2) three 100 × 100 × 100 mm cubic specimens for the 28-day compressive strength test.

To prepare the specimens, a laboratory mixer with a capacity of 15 L was utilized. Initially, all powder materials except quartz sand were added to the mixing bowl. The mixture was then dry-mixed for 2 min at a low speed of 100 rpm. Subsequently, quartz sand was added and mixed for an additional minute. While maintaining the mixer at low speed, water was slowly poured into the mixture for 30 s, and mixing continued for 3 min. Steel fibers were uniformly sprinkled by hand into the mixture until it reached a suitable viscosity and workability. The mixing speed was then increased to the maximum speed of 200 rpm and continued for 3 min before casting. For the PS groups, PS beads were added to the mixture 1 min after the steel fibers had been incorporated. The total mixing time was the same for all the groups.

Following the mixing procedure, the mixture was immediately poured into molds without any vibration. The molds were covered with a plastic sheet to prevent rapid water loss. After 24 h of casting, the specimens were demolded and then submerged into water for a 28-day curing at a curing temperature of 25 °C. The compressive tests and uniaxial tensile tests were conducted after the 28-day curing period.

**Figure 4:**
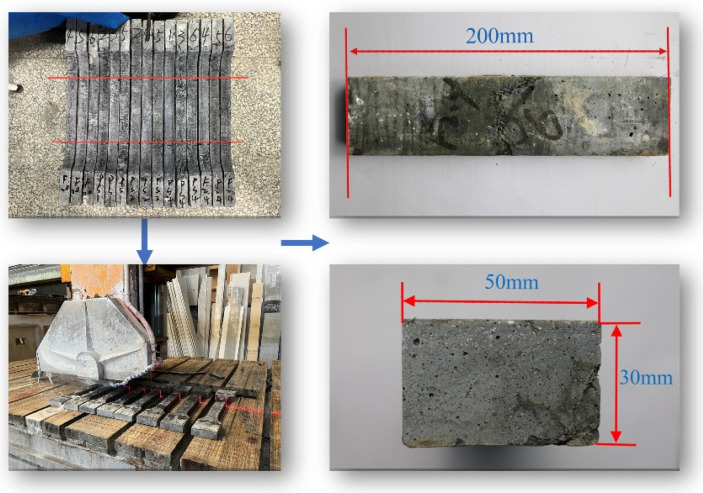
Specimens for X-ray CT scanning.

After the tensile tests, the uniaxial tensile specimens were cut into prisms for X-ray computed tomography (CT) scanning. This procedure aimed to establish a correlation between the tensile properties and the spatial distribution of the steel fibers and PS beads. The middle segment of the dog-bone specimens, measuring 30 × 50 × 200 mm and located within the range of linear variable displacement transducer (LVDT) measurements, was selected for CT scanning. The specimen with a tensile strength at the median value in each group was selected for CT scanning. In this study, the uniaxial tensile specimens F2.0-2, PS1-2, PS2-1, and PS4-1 were selected for CT scanning. The dimensions and preparation of the specimens used for CT scanning are displayed in Figure 4. This preparation method offers a more direct link between CT scanning tests and uniaxial tensile tests, eliminating the need to prepare additional specimens individually.

### 2.2. Uniaxial Tensile Test

**Figure 5:**
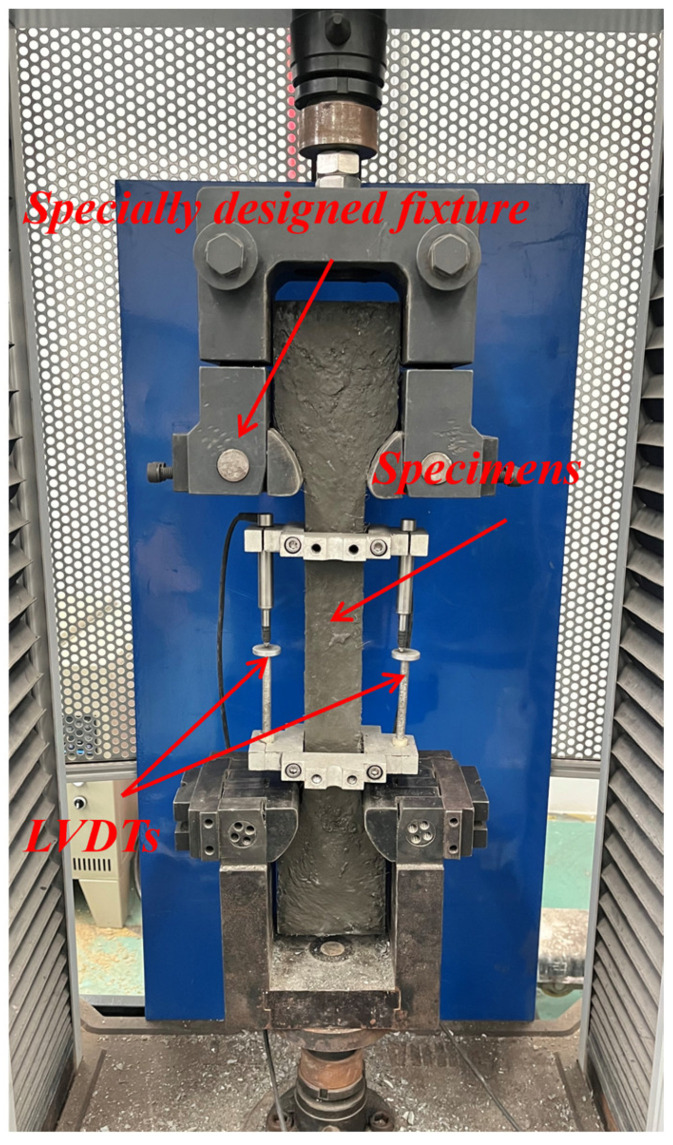
Setup for the tensile tests.

The setup for the tensile tests is depicted in Figure 5. A universal testing machine with a load capacity of 100 kN, operating in displacement control mode, was utilized for the uniaxial tensile tests. The tests comprised two stages: preloading and loading, with loading rates set at 1 mm/min and 0.3 mm/min, respectively. The tests commenced and concluded at 0.5 kN and 60% of the peak stress, respectively. To ensure pure tensile stress on the tensile specimens during tensile loading, a specially designed fixture was employed. Two high-precision linear variable displacement transducers (LVDTs) were employed to measure the elongation of the tensile specimens over a gauge length (L) of 200 mm. The LVDTs were affixed to a metal frame, which was secured onto the tensile specimens. The stress (σ) and average strain (ε) of each specimen used in the stress-strain curve needed to be determined using Equations (4) and (5).

(4) $$\varepsilon =(∆L_{1}+∆L_{2})/2L\times 100\%$$

(5) σ=F/A

where

- $∆L_{1},∆L_{2}$: displacement values recorded by the two LVDTs, $∆L_{1},∆L_{2}$;
- F: load applied by the testing machine;
- A: sectional area of tensile specimen (50 × 30 mm).

**Figure 6:**
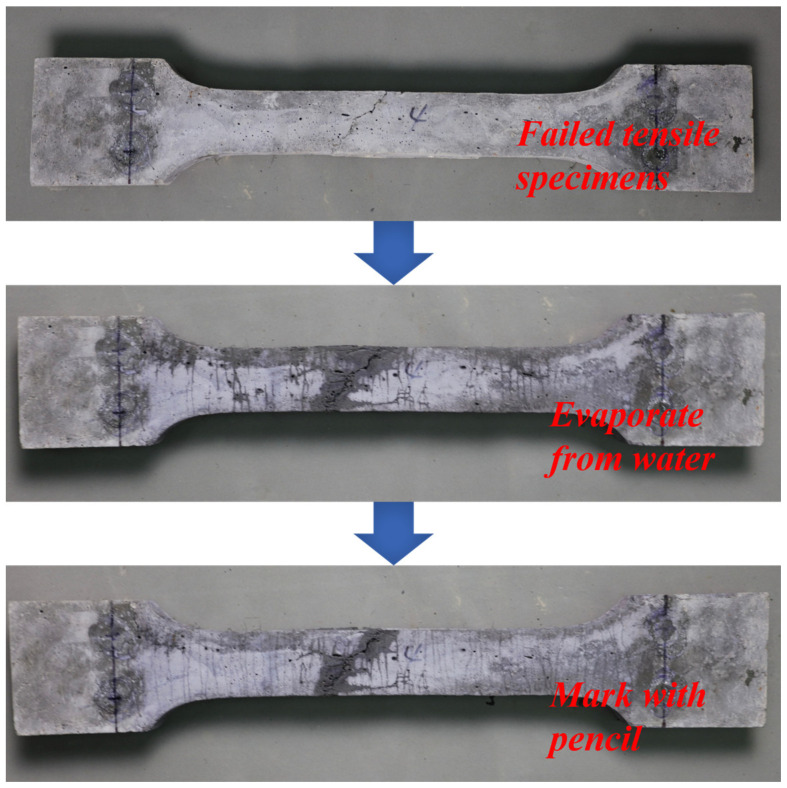
Treatment for visualization of microcracks after tensile tests.

To evaluate the cracking behavior of the UHPC matrix, the number of cracks and crack spacing needed to be determined. Since UHPC has excellent crack width control capabilities, most of the cracks formed during the tensile test are invisible to the naked eye (typically below 0.05 mm) [2]. After unloading, the cracks tend to close, making it difficult to visually discern the cracks. To visualize the cracks, special treatment, as shown in Figure 6, was performed on the tensile specimens after unloading. First, the specimens were dried in an oven at 70 °C for 12 h until the surface was completely dry. Subsequently, the specimens were immediately immersed in water. After 30 s of immersion, the specimens were removed from the water and allowed to dry in air. At this stage, the specimens were at a higher temperature compared to the water, causing rapid evaporation. The cracks, having absorbed more water, dried later than the remaining specimen’s surface, resulting in darker visible cracks. To enhance their visibility, a pencil was used to mark the cracks for easy recognition. Only cracks within the gauge length of the LVDTs were recorded and analyzed.

### 2.3. Compressive Test

In order to explore whether the compressive strength of UHPC decreases significantly after the addition of PS beads, the compressive strength of UHPC with different PS bead dosages was tested. The compressive strength of each specimen was measured according to GB/T 31387-2015 [40]. The 100 mm cube specimen was used for the test, three specimens for each mixture were tested, and average values and standard deviation on compressive strength were calculated. The test was carried out with a 30 t automatic testing machine, the loading speed of the experiment was 1.3 MPa/s, and the experiment was automatically stopped after the specimen was destroyed.

### 2.4. X-ray CT Test

In this study, CT scanning was conducted using an InspeXio SMX-225CT FPD HR Plus industrial CT scanner (Shimadzu, Kyoto, Japan). The scanner has the ability to process specimens with a maximum size of 400 mm in diameter and 300 mm in height, and the specimen platform can accommodate loads up to 12 kg. Equipped with a 1000 μA/225 kV/135 W X-ray tube, the CT scanner is capable of producing 2D images with a resolution of 4096 × 4096. To achieve higher image quality, a voltage of 170 kV and a current of 200 μA were selected for the X-ray source in this study.

**Figure 7:**
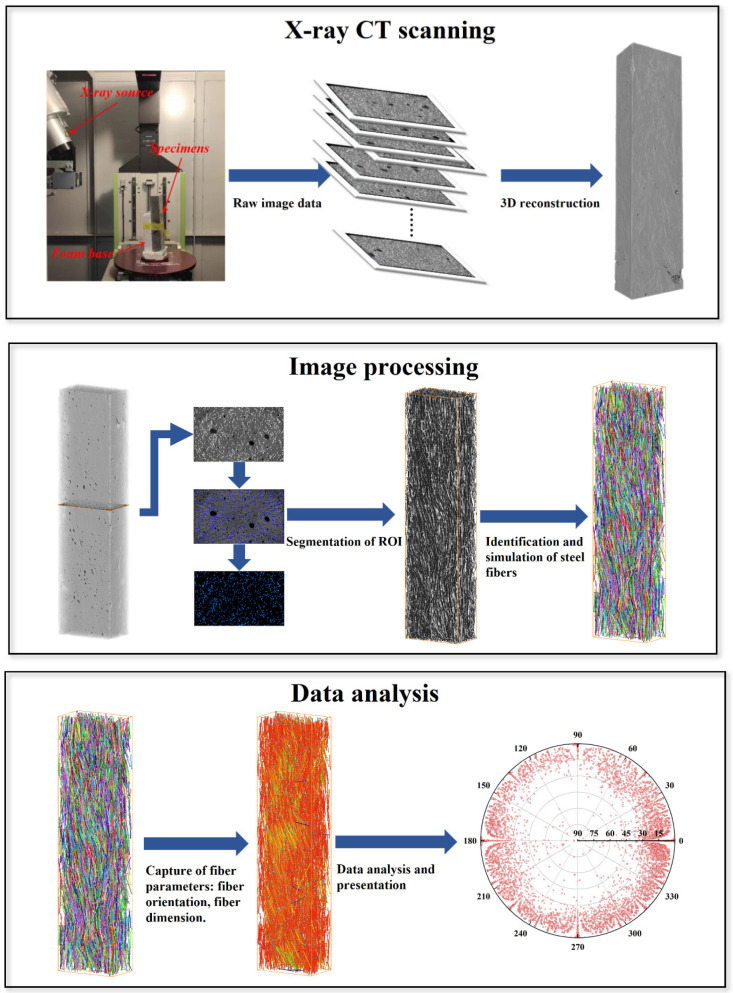
Workflow of processing CT scanning result.

As depicted in Figure 7, prior to CT scanning, each specimen was positioned on the rotating table of the device. To ensure stability during the scanning process, each specimen was secured with a foam base. Following the scanning procedure, the built-in software of the CT device generated 1354 2D raw images. These 2D images were then reconstructed into 3D images with a resolution of 148.5 μm, resulting in a voxel size of 148.5 × 148.5 × 148.5 μm. The grayscale information of the sample was preserved in the 2D images, where the grayscale values corresponded to the density of different components within the sample.

The exported images obtained from the CT scanner were imported into AVIZO 2022.2 software, a 3D visualization tool that has been widely used to analyze images obtained from CT scanning in previous studies [32,41,42]. The processing objectives in this study were as follows: extracting the flaw structures, including pores and PS beads, from the specimen and analyzing their sizes and 3D distributions; extracting the steel fibers and analyzing their distribution and orientation. The image processing workflow, illustrated in Figure 7, consisted of several steps. First, the image was segmented based on grayscale values to extract the region of interest. Next, denoising, smoothing, and other processing techniques were applied to the extracted region, and the effectiveness of the extraction process was evaluated by comparing the volume fraction of the extracted result with the actual volume fraction. Subsequently, data analysis was performed on the extracted construction using the built-in calculation tools in AVIZO to compute parameters such as porosity, pore size, and fiber orientation. Finally, the obtained parameters were exported for further analysis and processing.

## 3. Results

### 3.1. Compressive Strength

**Figure 8:**
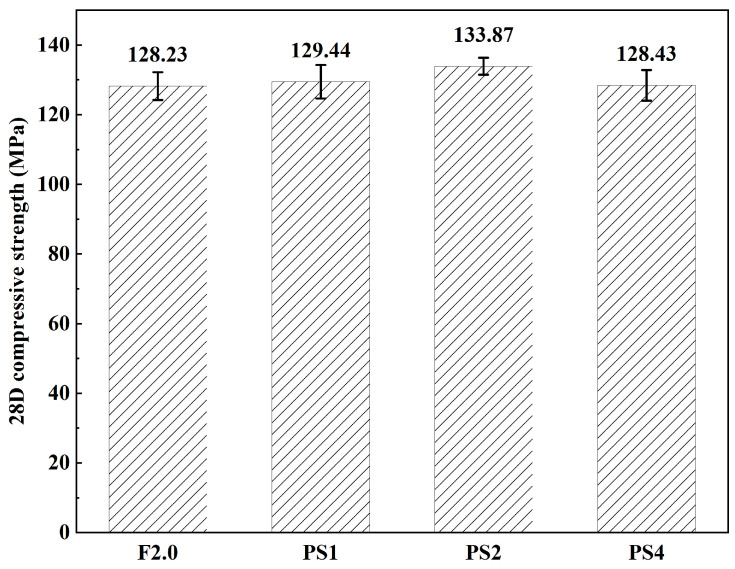
Compressive strength of each group.

Figure 8 illustrates the compressive strength of each group. The PS groups exhibited a slight improvement in compressive strength compared to the control group, with enhancements of 5%, 4.6%, and 0.2% observed for PS1, PS2, and PS4, respectively. These findings contradict the results reported in the literature [27], which indicated a decrease in compressive strength after incorporating artificial flaws into UHPC. Notably, the PS2 group exhibited the highest average compressive strength, reaching 133.87 MPa. As the dosage of PS beads increased, the compressive strength showed a trend of initially increasing and then decreasing. Overall, the fluctuation range of UHPC compressive strength is within 5%, and the dosages of PS beads in this study have no significant effect on it. The increase in the compressive strength of UHPC may be due to the significant reduction in the fiber clustering and the more uniform distribution of the steel fibers after the incorporation of PS beads, which will be discussed in Section 4.1.2.

### 3.2. Tensile Properties

**Figure 9:**
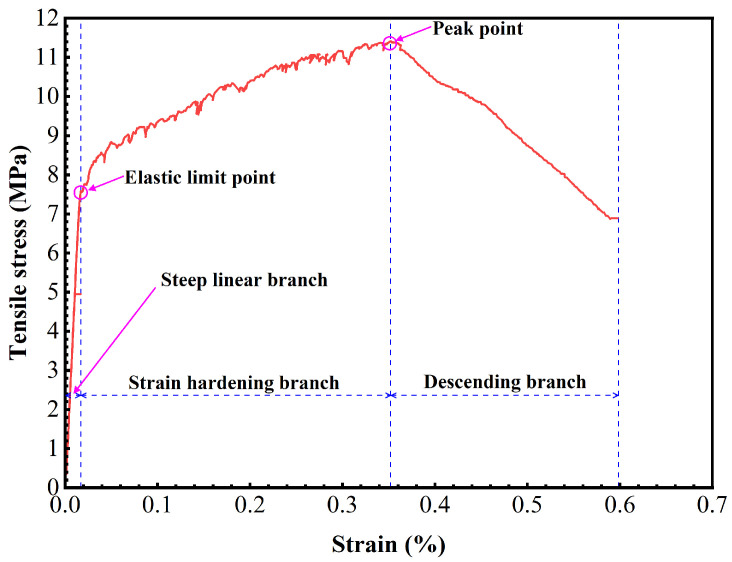
Typical stress–strain curve for strain-hardening UHPC.

In this study, all the specimens exhibited strain-hardening behavior. A typical stress-strain curve for strain-hardening UHPC is depicted in Figure 9. The curve can be divided into three regions: a steep linear branch, a strain-hardening branch, and a descending branch, separated by an elastic limit point ($f_{Ute}$, $\varepsilon_{Ute}$) and peak point ($f_{Utu}$, $\varepsilon_{Utu}$). The elastic limit corresponds to the peak value before the first significant drop in the steep linear branch, indicating the point where the first cracking occurs. The peak point represents the maximum stress value in the curve.

**Figure 10:**
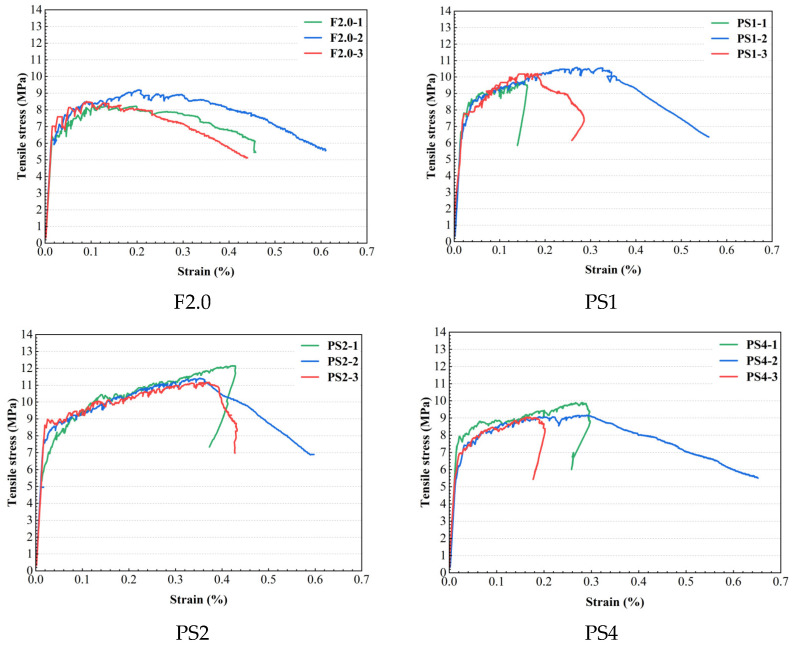
Strain–stress curves of all the specimens.

Figure 10 presents stress-strain curves for all tensile specimens cast in this study. In some stress-strain curves, a rebound in strain can be observed in the descending branch due to the presence of a major crack outside the gauge section. Table 3 provides detailed information, including the elastic tensile strength ($f_{Ute}$), elastic tensile strain ($\varepsilon_{Ute}$), ultimate tensile strength ($f_{Utu}$), ultimate tensile strain ($\varepsilon_{Utu}$), and strand-hardening ratio (${f_{Utu}/f}_{Ute}$). As shown in Figure 10 and Table 3, significant differences are observed among the different groups. The addition of PS beads significantly enhances the tensile performance of UHPC, as evidenced by the experimental results.

**Figure 11:**
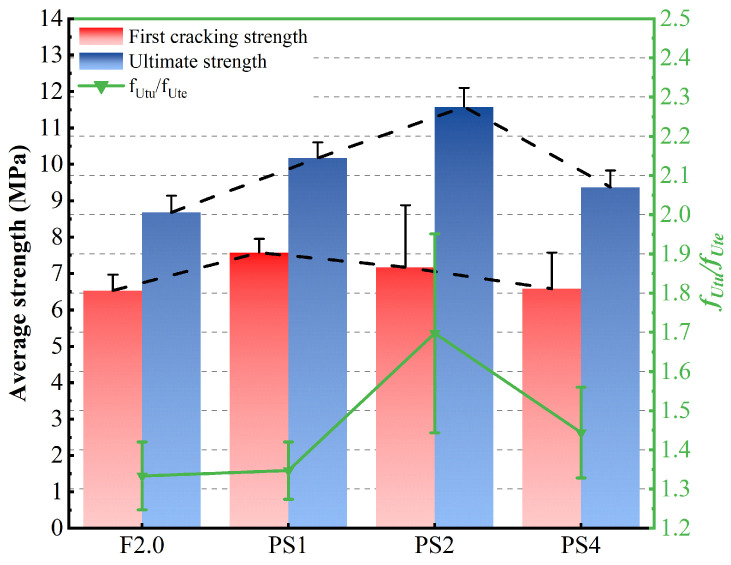
$f_{Ute}$, $f_{Utu}$, and ${f_{Utu}/f}_{Ute}$ of distinct groups.

Figure 11 displays the $f_{Ute}$, $f_{Utu}$, and strand-hardening ratio of all the groups. It is evident that incorporating PS beads into the UHPC matrix effectively enhances $f_{Utu}$. The average $f_{Utu}$ values of the PS1, PS2, and PS4 mixtures are 10.17 MPa, 11.58 MPa, and 9.37 MPa, respectively, corresponding to 117.2%, 133.4%, and 107.9% of the average $f_{Utu}$ of the F2.0 mixture. With an increase in PS bead dosage, the tensile strength initially increases until reaching a peak at a 2% volume fraction, after which it starts to decrease. The average $f_{Ute}$ values of PS1 and PS2 even show slight increases of 3.2% and 5.5%, respectively, compared to that of F2.0. However, with a further increase in PS bead content, the average $f_{Ute}$ of PS4 decreases by 3.8% compared to that of F2.0. Compared to $f_{Utu}$, the change in $f_{Ute}$ among different groups is more subtle. According to the latest recommendation issued by MCS-EPFL, the value of ${f_{Utu}/f}_{Ute}$ for UB (high strain-hardening UHPC) should be greater than or equal to 1.2. As shown in Figure 11, all the specimens in this study have a value of ${f_{Utu}/f}_{Ute}$ greater than 1.2. The strain-hardening ratio of specimen PS2-1 even reaches 2.29. The trend of ${f_{Utu}/f}_{Ute}$ is the same as that of $f_{Utu}$, increasing first and then decreasing with increasing PS bead dosage, peaking in the PS2 group with an average value of 1.74.

**Figure 12:**
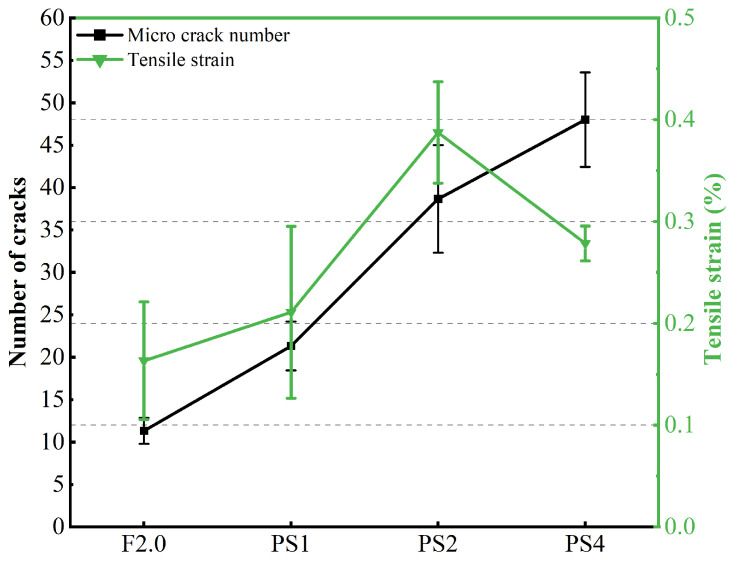
The tensile strain and microcrack number of distinct groups.

Compared to $f_{Utu}$, the change in ultimate tensile strain ($\varepsilon_{Utu}$) is more significant after incorporating PS beads. The average $\varepsilon_{Utu}$ values for the PS1, PS2, and PS4 mixtures are 0.2017%, 0.3820%, and 0.2418%, respectively, equivalent to 145.1%, 274.8%, and 174.0% of the value for the F2.0 mixture. As the PS bead dosage increases, $\varepsilon_{Utu}$ initially increases, reaching a peak at a 2% volume fraction, and then decreases (shown in Figure 12). Although the $\varepsilon_{Utu}$ value of the PS4 group is lower than that of the PS2 group, it is still much higher than that of the F2.0 group. Moreover, the difference between $\varepsilon_{Ute}$ and $\varepsilon_{Utu}$ shows an enhancement after incorporating PS beads, indicating that the strain-hardening behavior becomes more pronounced.

In contrast, Su-Tae Kang et al. [27] explored the effect of 2% volume fraction of plastic particles on the mechanical properties of UHPC with 1.5% steel fiber content in volume, and the test results showed that compared with the control group, the compressive strength of UHPC decreased by 4%, the $f_{Ute}$ and $\varepsilon_{Utu}$ of UHPC decreased by 5.6% and 5.5%, respectively, and the $f_{Utu}$ and ${f_{Utu}/f}_{Ute}$ of UHPC increased by 1.4% at 7.1%.

Table 4 presents detailed parameters of the microcracks that occurred within the gauge length of the LVDTs. Since these parameters are collected within the gauge length of the LVDTs, they provide a more direct reflection of their relationship with the uniaxial tension test results. The average crack spacing in the table represents the average distance between adjacent microcracks, calculated using Equation (6):

(6) $$x_{d}=L/n$$

where

- $x_{d}$: average crack spacing;
- L: the gauge length of the LVDTs;
- n: the number of microcracks within the gauge length of the LVDTs.

**Table 4:** Cracking parameters of specimens.

Specimen	Microcrack Number	Average	Average Crack Spacing (mm)	Average (mm)
F2.0-1	13	11	15.4	17.9
F2.0-2	11		18.2	
F2.0-3	10		20.0	
PS1-1	23	21	8.7	9.5
PS1-2	18		11.1	
PS1-3	23		8.7	
PS2-1	46	39	4.3	5.3
PS2-2	35		5.7	
PS2-3	35		5.7	
PS4-1	54	48	3.7	4.2
PS4-2	43		4.7	
PS4-3	47		4.3	

**Figure 13:**
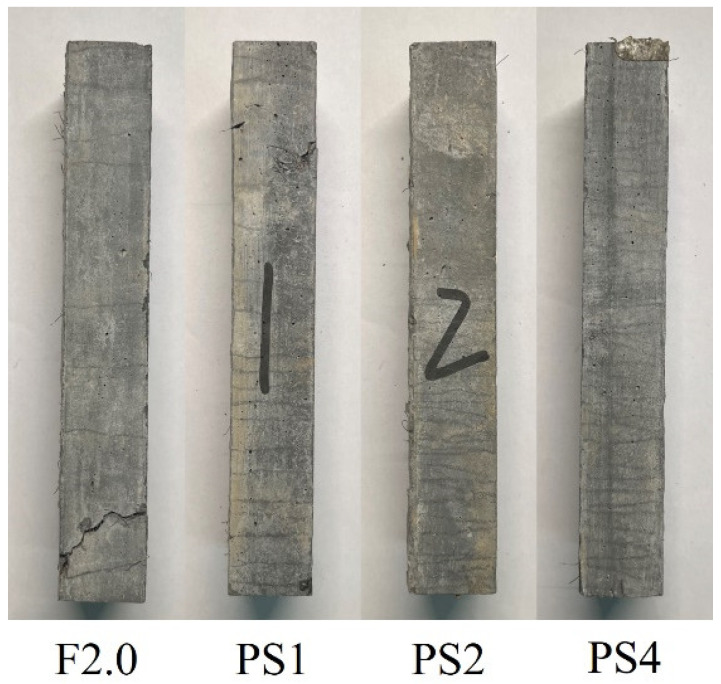
Cracking pattern of tensile specimens.

The average microcrack numbers for the F2.0, PS1, PS2, and PS4 groups are 11, 21, 39, and 48, respectively. The addition of PS beads to the UHPC matrix doubles the number of microcracks in the UHPC specimens, indicating a more pronounced multiple-cracking behavior during the tensile test. Figure 13 illustrates the cracking patterns observed in different groups. The PS groups exhibit closely spaced cracks, whereas the F2.0 group displays a significant crack-free area, which is consistent with the average crack spacing reported in Table 4.

### 3.3. CT Test Results

In this study, X-ray CT scanning technology was employed to scan the UHPC tensile specimens. By utilizing the three-dimensional visualization software AVIZO, the 2D images obtained from the scanner could be reconstructed into 3D images. The extracted three-dimensional images provided the basis for conducting quantitative analyses of the morphology, distribution, and other relevant information pertaining to the UHPC components. By analyzing these images, researchers gained valuable insights into the relationship between material structural characteristics and the mechanical behavior of the material.

#### 3.3.1. Flaw Distribution

The PS beads used as artificial flaws in the UHPC specimens had a significantly larger particle size compared to the maximum particle size of the UHPC powder. Additionally, they had a different density than the UHPC matrix. Therefore, achieving an even distribution of the PS beads throughout the UHPC matrix, without sinking, floating, or agglomerating, was crucial for their role as artificial flaws.

**Figure 14:**
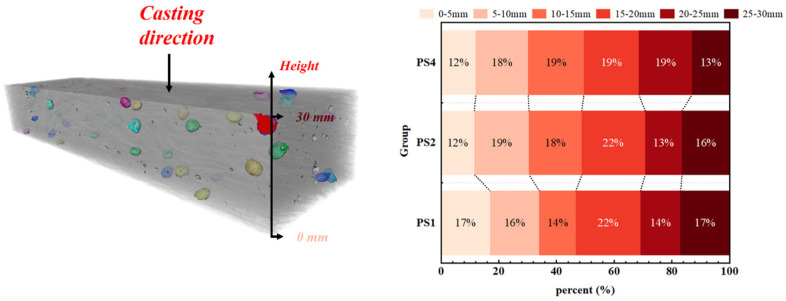
PS bead distribution in height.

A total of 85, 164, and 331 PS beads were extracted from the PS1, PS2, and PS4 groups, respectively. The distribution of PS beads throughout the height of the specimen was analyzed, as shown in Figure 14. The specimen height was divided into six 5 mm intervals, with darker colors indicating higher heights. The percentage in the figure represents the ratio of the number of PS beads in a specific interval to the total number of PS beads extracted from that specimen. The centroid position height of each PS bead was used to determine its interval. The results showed that in the PS1, PS2, and PS4 groups, 47%, 49%, and 49% of the PS beads were located in the lower half of the specimen, respectively. The number of PS beads in the upper half (15–30 mm) and lower half (0–15 mm) was nearly equal for all specimens. As the PS bead content increased, the distribution of PS beads throughout the height of the specimen became more uniform.

**Figure 15:**
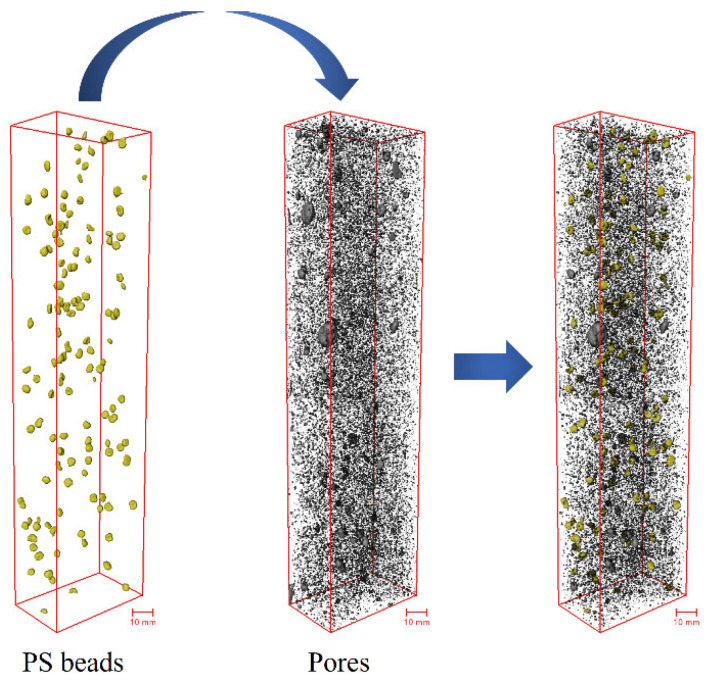
Flaw structure in UHPC.

**Figure 16:**
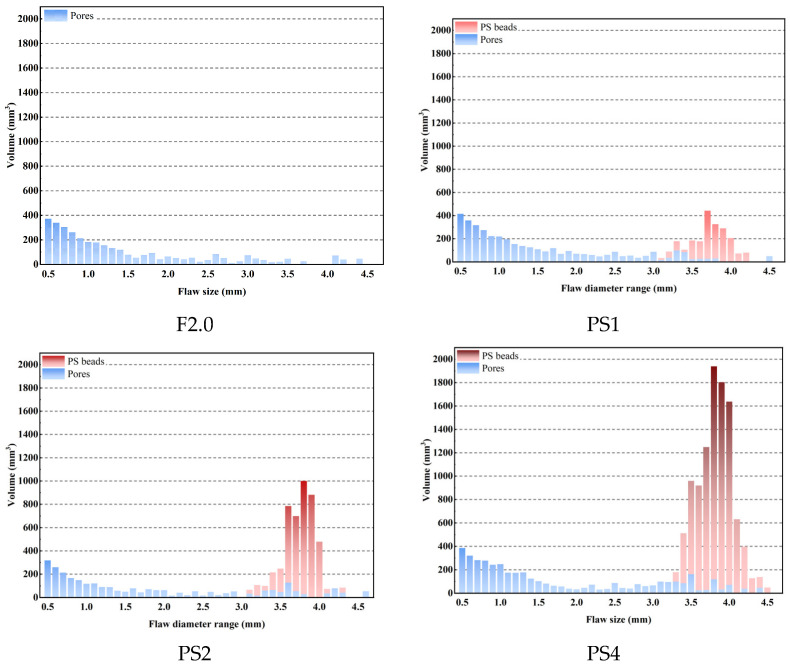
Flaw distribution of UHPC matrix.

Studies [18,25,43] have shown that only when the flaw size is larger than $C_{mc}$, the flaws will contribute to the tensile multiple cracking of fiber-reinforced cementitious materials. ShuXing Wang et al. [21] proposed that it is reasonable to approximate $C_{mc}$ as the maximum pore size at the crack interface of the failed uniaxial tension specimens. The maximum pore diameters of PS1, PS2, and PS4 are 4.52 mm, 4.68 mm, and 4.47 mm, respectively, which are close to the diameters of the PS beads used in this study. As shown in Figure 15, after adding PS beads, the flaw structure of the UHPC matrix can be regarded as the superposition of PS beads and inherent pore flaws. The PS beads had little deviation in particle size, which was approximately 4 mm in diameter. Therefore, this section focuses on the changes in flaw structure with equivalent diameters larger than 0.5 mm. Figure 16 shows the pore flaw distributions of PS1, PS2, and PS4 and the flaw distributions after adding PS beads. The horizontal axis represents the equivalent diameter of flaws, divided into intervals of 0.1 mm, and the vertical axis represents the total volume of flaws in that equivalent diameter interval. The results show that adding PS beads can significantly change the flaw structure, especially the large flaw distribution of the UHPC matrix, and the change is more significant with the increasing dosage of PS beads.

#### 3.3.2. Fiber Orientation

After importing the CT scan results into the 3D visualization software AVIZO, image segmentation was performed to extract the steel fibers. The X-Fiber module in the software was used to process the obtained steel fiber images. There were more than 7000 fibers in each CT specimen, making the analysis of their orientation information challenging. The software processing allowed for the identification of each fiber and its replacement with a simulated fiber, enabling the construction of a fiber network model. The simulated fiber was designed to mimic the length, orientation, diameter, and other characteristics of the actual fiber. The effectiveness of the fiber simulation can be observed in Table 5.

**Figure 17:**
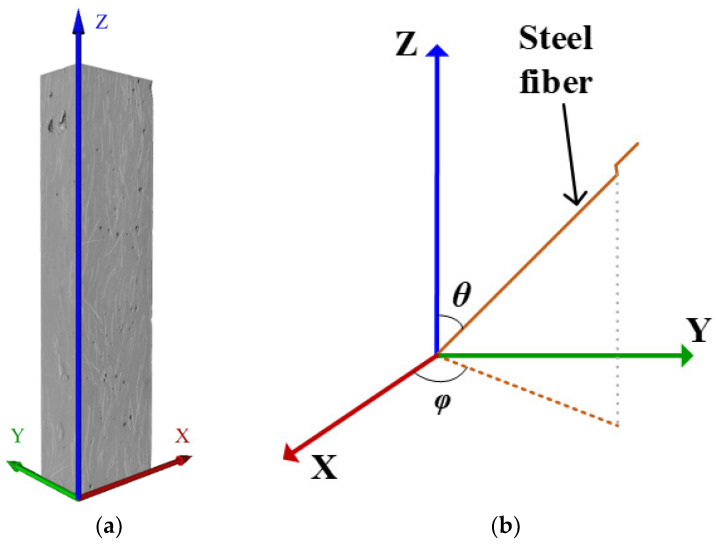
(**a**) Coordinate system; (**b**) definition of the orientation angles θ and φ.

For the CT specimens of the F2.0, PS1, PS2, and PS4 groups, a total of 7342, 7233, 7109, and 6982 steel fibers were extracted using AVIZO software, respectively, which closely matches the estimated numbers. The average lengths of the simulated fibers were recorded as 13.52 mm, 13.00 mm, 12.78 mm, and 13.30 mm. Notably, the simulated fiber length was shorter than the actual length because of excluding the hooked portion of the hooked fiber when setting the simulation parameters. The average length of the simulated fiber represented the middle straight section of the hooked fiber. In summary, the fiber simulation process successfully matched the actual situation, indicating a satisfactory simulation result. By utilizing the fiber network constructed with simulated fibers, it became possible to obtain the actual distribution and orientation information of the steel fibers within the UHPC specimens. A coordinate system, as depicted in Figure 17, was established to represent the fiber orientation. The determination of fiber orientation angles (θ and φ values) followed the same methodology described in the literature [31,33].

**Figure 18:**
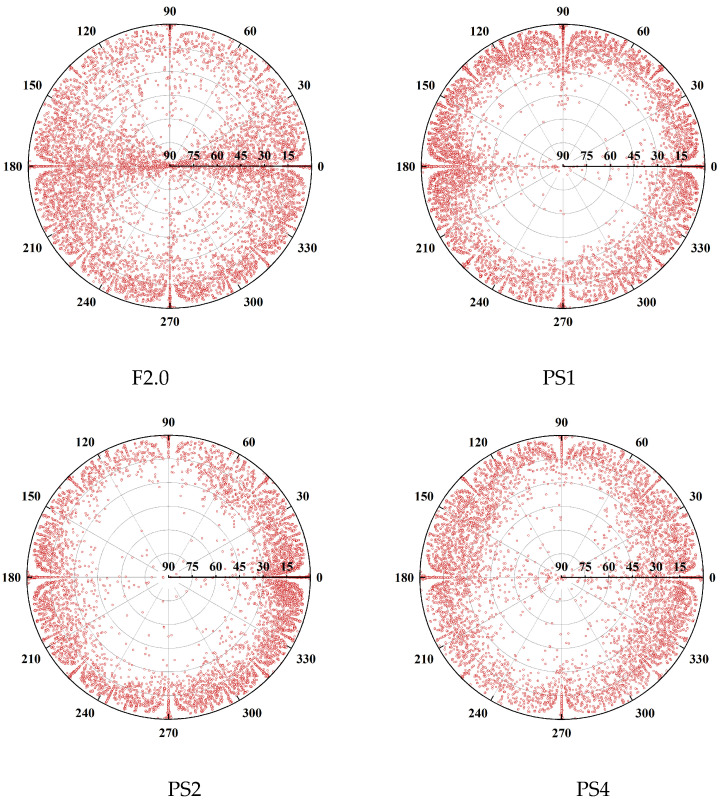
Scatter plot of fiber orientation.

The fiber orientation distribution of all the specimens is plotted in Figure 18, revealing significant changes after the addition of PS beads. In the scatter plot, the angular direction and the radius direction represent the φ value and θ value of the fiber, respectively. It can be observed that the distribution in the angular direction is relatively uniform. However, in all specimens, the fibers tend to concentrate at angles of φ = 0°, 45°, 90°, 135°, 180°, 225°, 270°, and 315°. This phenomenon is attributed to the wall effect, wherein fibers near the edges of the mold align with the mold’s orientation [44,45,46,47].

To further investigate the distribution of fibers along the uniaxial tension direction (Z-axis direction), a parameter called orientation number $\eta_{\xi }$ is introduced. Orientation number $\eta_{\xi }$ has been commonly used in previous studies [48,49] to describe the fiber distribution in a specific direction. Its definition is as follows.

(7) $$\eta_{\xi }=\frac{1}{N}\sum_{i=1}^{N}cos\theta_{i}^{\xi }$$

In the formula, $\eta_{\xi }$ represents the orientation number with respect to the ξ axis. It is calculated by dividing the number of fibers (N) by the sum of the cosine values of the angles ($\theta_{i}$) between each fiber and the ξ axis.

The corresponding $\eta_{Z}$ values for each specimen are provided in Table 6. The $\eta_{Z}$ values for the specimens with PS beads (PS1, PS2, and PS4) are significantly higher than those for the F2.0 group. Additionally, there is a trend of increasing and then decreasing $\eta_{Z}$ values with increasing PS bead content, peaking at 2% PS content.

**Figure 19:**
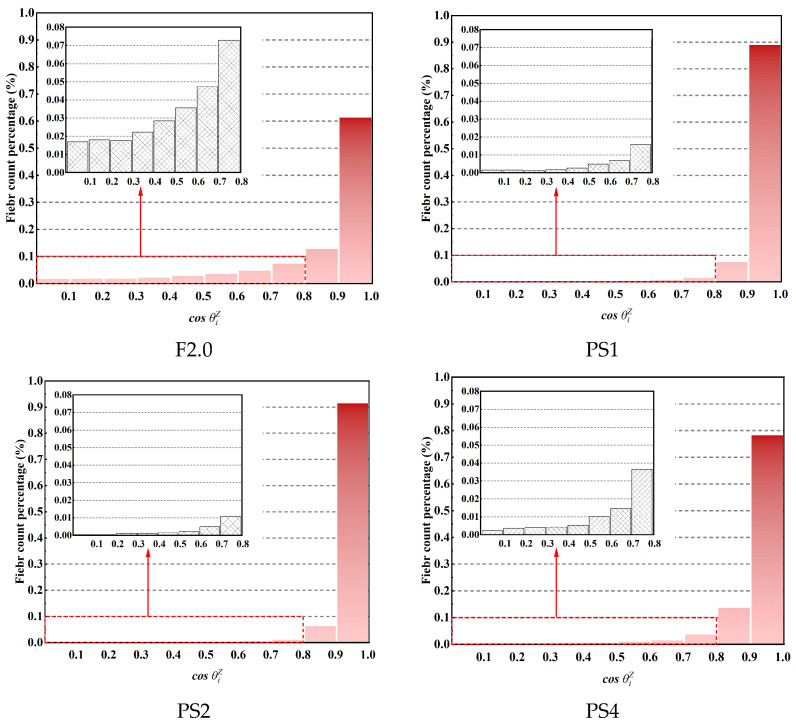
Distribution of value of $cos\theta_{i}^{Z}$ for each fiber in all the groups.

Figure 19 illustrates the distribution of $cos\theta_{i}^{Z}$ values of steel fibers for all specimens. The horizontal axis represents $cos\theta_{i}^{Z}$, which is divided into ten intervals from 0 to 1 with an interval of 0.1. The vertical axis represents the percentage of fibers within each interval relative to the total number of fibers. The distribution of $cos\theta_{i}^{Z}$ values for all specimens shows a similar trend; as $cos\theta_{i}^{Z}$ increases, the corresponding number of fibers also increases. Most of the fibers have $cos\theta_{i}^{Z}$ values within an interval of [0.8–1.0]. To provide more clarity for $cos\theta_{i}^{Z}$ values less than 0.8, a local magnification is applied. It is observed that the distribution of fibers with $cos\theta_{i}^{Z}$ values less than 0.8 exhibits more significant differences among the different groups compared to the distribution of fibers with $cos\theta_{i}^{Z}$ values greater than 0.8. In the F2.0, PS1, PS2, and PS4 specimens, the proportions of fibers with $cos\theta_{i}^{Z}$ values less than 0.8 (corresponding to $\theta_{i}^{Z}$ greater than 36.9 degrees) are 25.94%, 3.57%, 2.28%, and 5.87%, respectively. Adding PS beads effectively reduces the number of fibers with large $\theta_{i}^{Z}$ angles, indicating a decrease in the number of fibers that could be detrimental to uniaxial tensile performance.

## 4. Discussion

### 4.1. Effect of PS Beads on the Material Structures of UHPC

#### 4.1.1. Artificial Flaw in UHPC Matrix

Changes in the internal material structure led to variations in mechanical performance. After the incorporation of PS beads, UHPC exhibited significant multiple-cracking behavior. In comparison to the F2.0 group, the crack count increased from 11 to 21, 39, and 48 for the PS1, PS2, and PS4 groups, respectively. Additionally, the crack spacing decreased from 17.9 mm to 9.5 mm, 5.3 mm, and 4.2 mm. Importantly, the crack count used in this study does not represent the actual number of cracks but rather employs a special treatment method outlined in Section 2.2 for all specimens. Although this crack count may not be highly precise, it served as an acceptable measure for assessing the cracking behavior within different groups of UHPC matrices under tensile stress conditions. The experimental results demonstrated that the addition of PS beads in UHPC led to an increased number of cracks. The generation of more cracks implied a significant reduction in crack width under the same strain conditions. In fact, during the strain-hardening stage of the experiment, no visible cracks were observed on the surfaces of any specimens. Based on the estimated crack counts used in this study, the average crack width ($\varepsilon_{Utu}\times L$/crack counts) before strain softening for the F2.0 group was approximately 0.025 mm, while for the PS2 group, it should be smaller than 0.019 mm, below the naked-eye resolution of 0.05 mm for crack detection. Improved crack control capability enhanced the durability of structures and facilitated the potential self-healing ability of UHPC [50,51,52].

Although the PS1 and PS2 groups exhibited more pronounced multiple-cracking behavior compared to the F2.0 group after the addition of PS beads, their first cracking strength was slightly improved. The matrix cracking strength is sensitive to the flaw size within the matrix, where it reaches a lower bound rather than decreasing infinitely as the flaw size increases [24,25]. However, the presence of fibers influences the first cracking strength of UHPC. The first cracking strength of UHPC can be estimated with the following equation [53]:

(8) $$\sigma_{c}=\sigma_{mc}V_{m}+\eta_{l}\eta_{\theta }\sigma_{f}\varepsilon_{c}V_{f}$$

In Equation (8), the left-hand term, $\sigma_{mc}V_{m}$, represents the influence of the fiber-free matrix on the cracking strength of UHPC. The right-hand term, $\eta_{l}\eta_{\theta }\sigma_{f}\varepsilon_{c}V_{f}$, represents the effects of fibers on the cracking strength of UHPC, including the fiber orientation, fiber content, and fiber aspect ratio. When the flaw size in the UHPC matrix increases, it leads to a decrease in $\sigma_{mc}V_{m}$. On the other hand, when the fiber orientation becomes closer to the tensile stress direction, it results in an increase in $\eta_{l}\eta_{\theta }\sigma_{f}\varepsilon_{c}V_{f}$.

Although the flaw size within the UHPC matrix is significantly altered in the PS1-2 and PS2-1 groups after the addition of PS beads, the orientation numbers increase by 13.1% and 14.5% compared to the F2.0 group, respectively. When the dosage of PS beads is relatively low, the change in fiber orientation dominates the variation in cracking stress. The orientation number of the PS4-1 group decreases compared with that of PS2-1 but is 9.5% higher than that of F2.0-3, and the first cracking strength is 11.5% lower than that of F2.0-3, indicating that the increased flaw size here primarily influences the first cracking strength. The first cracking strength of UHPC is influenced by the combined effect of flaw size variation and changes in fiber orientation.

#### 4.1.2. Interaction with Steel Fibers

In this study, the intention of introducing PS beads was to decrease the cracking strength of the matrix and therefore enhance the tensile performance. However, unexpectedly, the addition of PS beads resulted in a significant increase in the tensile strength of UHPC, which was not reported in previous studies. The ultimate tensile strength of UHPC is determined by the fiber-bridging ability, which is influenced by the fiber parameters ($V_{f}L_{f}{/d}_{f}$), bond strength (τ), and fiber orientation [6,8,44,49]. In this study, the fiber content of all the groups was 2%. The PS beads had hydrophobic surfaces and hardly reacted chemically with the matrix, so it can be assumed that there was no significant difference in fiber parameters and fiber–matrix bond strength among all specimens. To obtain the fiber orientation parameters, the X-ray CT results of the specimens were analyzed in Section 3.3.2, and the results showed a significant change in fiber orientation distribution after adding PS beads. Compared to the F2.0 group, the fibers in the PS group tended to align more along the Z-axis, which is parallel to the direction of tensile stress in the axial tensile test. The significant change in steel fiber orientation is the reason for the improvement in UHPC tensile strength. This result was not recorded in any previous studies.

**Figure 20:**
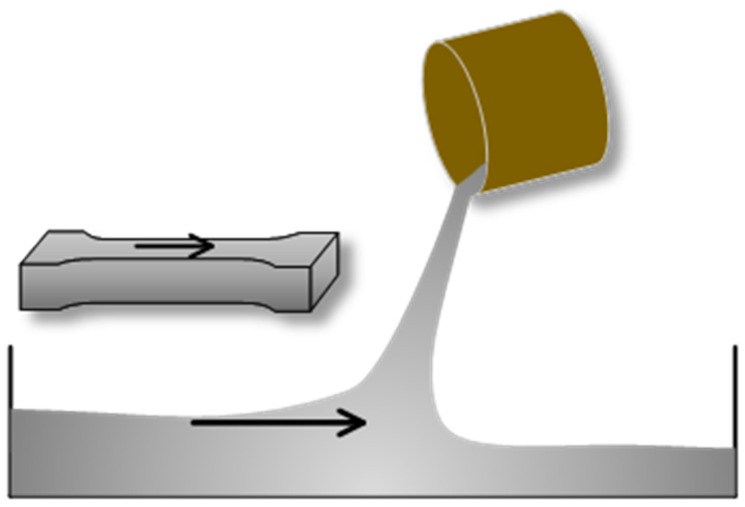
Casting method.

Fiber orientation is related to the fiber content, fiber shape, matrix fluidity, and UHPC casting method [46,48]. In this study, the casting method used is shown in Figure 20, where the casting direction is parallel to the length direction of the dog-bone specimens, which is also parallel to the direction of tensile stress in the specimen. In addition to the addition of PS beads, the composition of the PS group and the F2.0 group was identical, and under the same casting method, the fiber orientation in the PS group changed significantly compared to that in the F2.0 group. This is a result of the interaction between PS beads and fibers.

**Figure 21:**
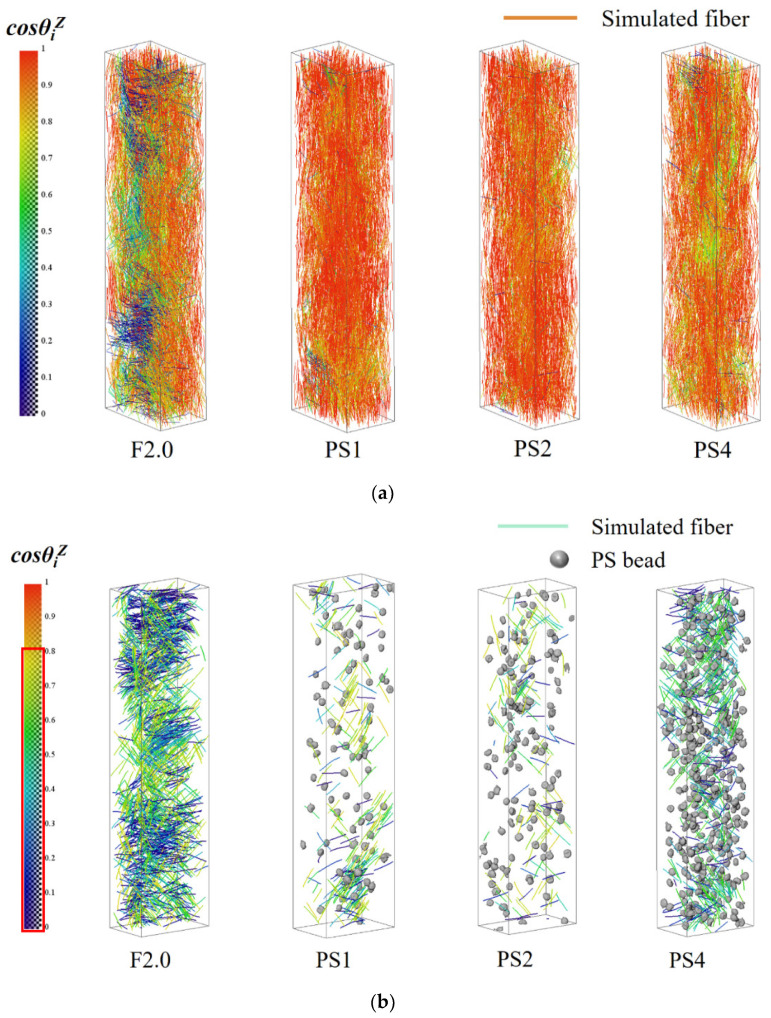
Three-dimensional visualization of steel fibers and PS beads: (**a**) all the simulated fibers; (**b**) PS beads and the simulated fibers with $cos\theta_{i}^{Z}$ values less than 0.8.

The interaction between PS beads and fibers can occur in both the mixing and casting processes. During the mixing process, the spherical PS beads are uniformly distributed in the UHPC matrix, which may facilitate a better dispersion of steel fibers and reduce fiber clustering. Figure 21 depicts the reconstruction of steel fibers and PS beads, where the color of the fibers corresponds to their cosine values. Figure 21a shows the reconstruction of steel fibers, while Figure 21b shows the reconstruction of PS beads and steel fibers with $cos\theta_{i}^{Z}$ values less than 0.8, along with PS beads. The F2.0 and PS1 groups showed localized fiber clustering, while in the PS2 and PS4 groups, there was almost no fiber clustering. The phenomenon of fiber clustering disappeared with the increase in PS beads, which played the role of a stirrer during the mixing process, assisting in more uniform fiber dispersion.

**Figure 22:**
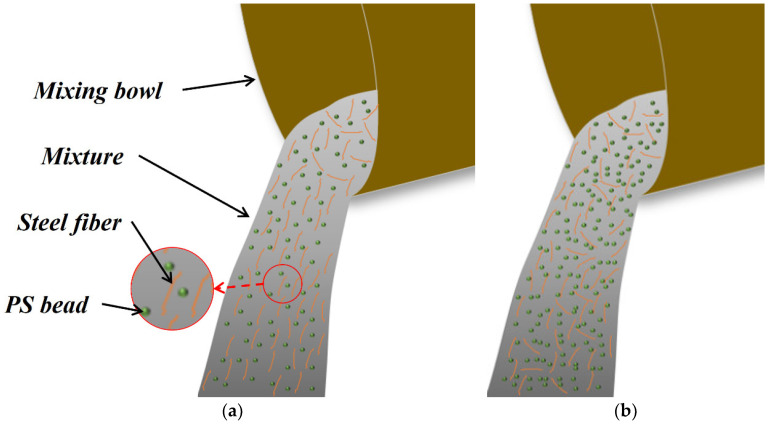
Fiber distribution during casting: (**a**) moderate content of PS beads; (**b**) excessive content of PS beads.

**Figure 23:**
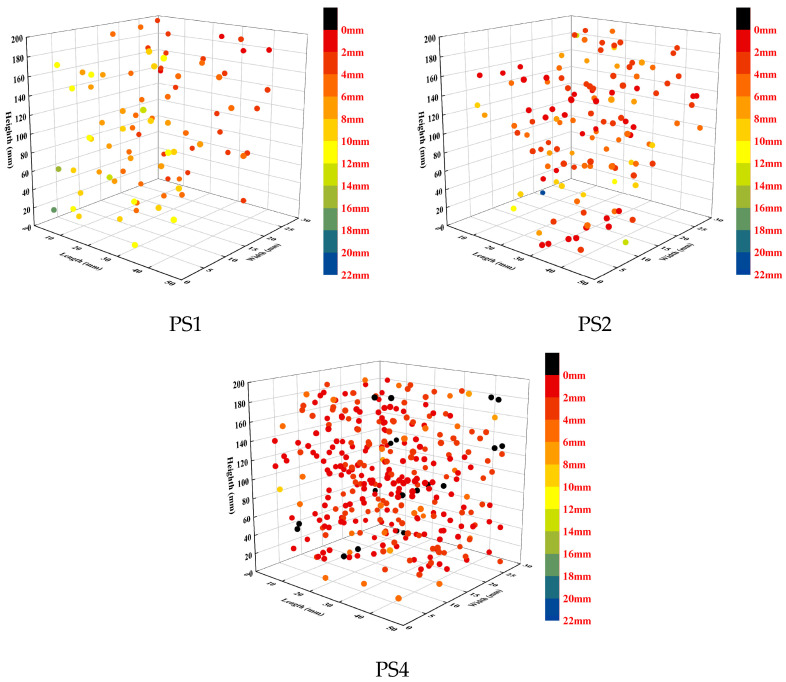
Closest distances between PS beads.

Notably, the $\eta_{Z}$ values showed an initial increase followed by a decrease with the increase in PS bead dosage. The fiber orientation distribution map (Figure 18) also indicates that the fiber orientation in the PS2 group was the most concentrated, while the PS1 and PS4 groups exhibited more dispersed orientations. Figure 21b shows that the F2.0 and PS1 groups had an obvious localized clustering of fibers, while the fibers in the PS2 and PS4 groups were evenly distributed between the PS beads without clustering. During the transfer of the UHPC mixture from the mixing bowl to the mold, the fibers tended to align along the flow direction of the fresh UHPC by rotation and displacement [44,45,46,47,54,55], which would be restricted due to the more limited space between PS beads as the dosage of PS beads increases (shown in Figure 22). The spatial coordinates of the PS beads were imported into MATLAB to calculate the distance between each bead and its nearest neighboring PS bead. The resulting distances are shown in Figure 23. In the plot, the position and color of each bead represent its centroid position and distance between that bead and its nearest neighboring bead (centroid distance minus the average diameter of the PS beads), respectively. The average spacings between adjacent PS beads in the PS1, PS2, and PS4 groups are 6.42 mm, 4.98, and 3.83 mm, respectively. In the PS4 group, some PS beads were observed to be almost in contact with each other (black beads). As shown in Figure 22, during the casting process, fibers with larger $\theta_{i}^{Z}$ values cannot redistribute along the casting direction. As a result, the $\eta_{Z}$ value in the PS4 group decreased compared to that in the PS2 group.

The change in $\eta_{Z}$ with the addition of PS beads is the result of an interaction between the two effects of PS beads acting as a stirrer and a limiter. In summary, there is an optimal volume fraction of PS beads. As the dosage of PS beads increases, more PS beads act as mixing stirrers, resulting in a reduction in the fiber clustering phenomenon. When it exceeds the optimal volume fraction, the poor space between PS beads restrict fiber rotation and displacement to orient along the casting direction during the casting process. In this study, the optimal volume fraction of PS beads is 2%.

### 4.2. The Correlation between Fiber Orientation and Tensile Properties

According to previous studies, the tensile strength of UHPC can be predicted using Equation (9) [56]:

(9) $$f_{Utu}=f_{Utm}\left(1-V_{f}\right)+\eta_{\theta }V_{f}\tau_{f}\frac{l_{f}}{d_{f}}$$

where

- $f_{Utu}$: the tensile strength of the UHPC;
- $f_{Utm}$: the strength of the matrix without fibers;
- $V_{f}$: the fiber volume fraction;
- $\eta_{\theta }$: the fiber orientation parameter;
- $\tau_{f}$: the interfacial bond strength between the fiber and the matrix;
- $\frac{l_{f}}{d_{f}}$: the aspect ratio of the fiber.

When other factors are constant, the fiber orientation is the determining factor for the $f_{Utu}$ of UHPC. In this study, the coefficient of variation (CV) for f_Utu_ of three specimens in each group is significantly less than 0.15, specifically 0.04, 0.03, 0.04, and 0.04, indicating good consistency in each group. Therefore, it can be assumed that the fiber orientation distribution is similar among the three specimens in a same group. It is acceptable to select the specimen with a median strength from the three specimens to represent the group for X-ray CT scanning.

**Figure 24:**
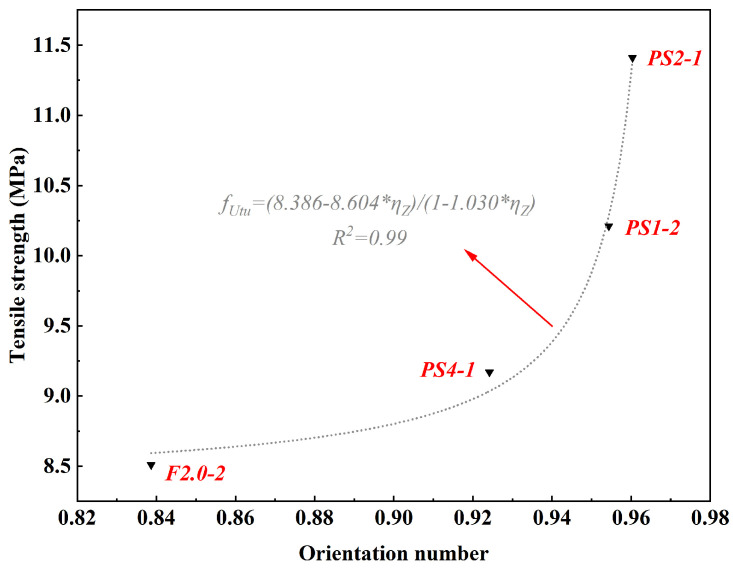
Relationship between orientation number and tensile strength.

In this study, the fiber orientation was characterized using the orientation number $\eta_{Z}$, and a scatter plot (Figure 24) was created to illustrate the relationship between $\eta_{Z}$ and the tensile strength of UHPC ($f_{Utu}$). It was found that the orientation number is related to the tensile strength of UHPC as follows:

(10) $$f_{Utu}=(8.386-8.064*\eta_{Z})/(1-1.030*\eta_{Z})$$

This relationship can be further simplified as follows:

(11) $$f_{Utu}=7.829+\frac{0.557}{1-1.030*\eta_{Z}}(R^{2}=0.99)$$

With an $R^{2}$ value of 0.99, the fitted results exhibit a strong correlation with the experimental results.

## 5. Conclusions

In this study, spherical PS beads were incorporated into a UHPC matrix, leading to significant changes in the flaw structure of the UHPC matrix and a notable improvement in its tensile performance. X-ray CT scanning of the UHPC tensile specimens was performed, and the structure of fibers and PS beads in UHPC was reconstructed using the three-dimensional visualization software AVIZO. The changes in internal material structure were used to explain the improvement in UHPC tensile properties. Based on the experimental results, the following conclusions were drawn:

1. The tensile properties of ultrahigh performance concrete (UHPC) were investigated by testing stress–strain curves for different PS (polystyrene) bead dosages (0%, 1%, 2%, 4% in volume fraction). The addition of PS particles significantly improved the tensile performance of UHPC. As the PS bead dosage increased, the tensile behavior exhibited an initial increase followed by a decline. The optimal tensile performance was achieved when the PS particle volume fraction was 2%, resulting in a 33.4% increase in the ultimate tensile strength, a 174.8% increase in the ultimate tensile strain, and a sharp increase in the strain-hardening ratio from 1.33 to 1.74 compared to the control group.
2. X-ray CT scanning was conducted, and its results were used to reconstruct the 3D structure of UHPC and analyze its internal defects, including pores and PS particles. The analysis revealed that PS beads were uniformly dispersed within the UHPC matrix, significantly altering the flaw size distribution. PS particle sizes were concentrated between 3.5 mm and 4.5 mm.
3. Based on the CT scan results, a distribution model for steel fibers was constructed, and their orientation was quantitatively analyzed. The addition of PS beads reduced steel fiber clustering, especially at a moderate PS bead dosage (volume fraction ≥ 2%). Proper PS particle dispersion facilitated uniform steel fiber distribution during the mixing procedure. However, excessive PS particle content (volume fraction ≤ 4%) hindered steel fiber alignment due to smaller spacing between adjacent PS beads, limiting their orientation along the casting direction. Changes in steel fiber spatial distribution were the primary cause of variations in UHPC tensile behavior.
4. Compressive strength tests were conducted on UHPC samples. Surprisingly, the addition of PS particles did not significantly reduce UHPC’s compressive strength; in fact, there was a slight increase (within 5%). This enhancement may be attributed to PS particles reducing steel fiber clustering, resulting in more uniform fiber distribution.
5. Limitations and Future Research: Despite these findings, there are limitations to this study. Further investigation is needed to understand the interaction between PS particles and steel fibers, especially during the mixing and casting processes. While this paper focused on UHPC’s tensile behavior, a deeper exploration of compressive strength changes and related mechanisms is warranted. Additionally, exploring the feasibility of using other industrial waste materials as substitutes for PS beads in this modification method is recommended.

## Figures and Tables

**Table 1:** Mix proportions of all the groups.

Type	F2.0	PS1	PS2	PS4
Cement	1			
Water	0.251			
Silica fume	0.214			
Mineral powder	0.214			
Quartz sand	1.064			
Superplasticizer	0.012			
Steel fiber in Vol (%)	2			
PS beads in Vol (%)	0	1	2	4

**Table 2:** Properties of steel fiber.

Fiber Shape	Length/mm	Diameter/mm	Aspect Ratio	Tensile Strength/MPa
Hooked fiber	16	0.2	80	2500

**Table 3:** Tensile properties of specimens.

No.	$f_{Ute}$ /MPa	Average (CV)	$\varepsilon_{Ute}$	Average (CV)	$f_{Utu}$ /MPa	Average (CV)	$\varepsilon_{Utu}$	Average (CV)	$f_{Utu}/f_{Ute}$	Average (CV)
F2.0-1	6.4	6.53 (0.05)	150	146.67 (0.06)	8.32	8.68 (0.04)	1223	1390 (0.34)	1.30	1.33 (0.09)
F2.0-2	6.18		135		9.2		2042		1.49	
F2.0-3	7.02		155		8.51		905		1.21	
PS1-1	6.68	6.74 (0.07)	149	161.00 (0.08)	9.73	10.17 (0.03)	1512	2017 (0.25)	1.46	1.52 (0.09)
PS1-2	6.24		155		10.58		2705		1.70	
PS1-3	7.31		179		10.21		1834		1.40	
PS2-1	5.3	6.89 (0.16)	120	154.00 (0.16)	12.16	11.58 (0.04)	4226	3820 (0.08)	2.29	1.74 (0.22)
PS2-2	7.58		171		11.41		3522		1.51	
PS2-3	7.78		171		11.16		3713		1.43	
PS4-1	7.36	6.35 (0.12)	157	154.00 (0.07)	9.9	9.37 (0.04)	2664	2418 (0.22)	1.35	1.49 (0.09)
PS4-2	5.47		140		9.17		2905		1.68	
PS4-3	6.22		165		9.05		1687		1.45	

**Table 5:** Information of the simulated fibers.

Specimen	Fiber Count	Average Length of Simulated Fiber (mm)
F2.0	7342	13.52
PS1	7233	13.00
PS2	7109	12.78
PS4	6982	13.30

**Table 6:** Orientation number η_Z_ of the specimens.

Specimen	$\eta_{Z}$	Deviation
F2.0	0.838652	0.227406507
PS1	0.954344	0.08717
PS2	0.960323	0.069437
PS3	0.92419	0.121344

## Data Availability

The data presented in this study are available within the article.
